# Supplementary material for: Identification of the EH CRISPR‐Cas9 system on a metagenome and its application to genome engineering
Source: Microb Biotechnol. 2023 Apr 25;16(7):1505–23. doi: 10.1111/1751-7915.14266 (PMC10281378; doi:10.1111/1751-7915.14266)
Supplement: Supplementary file 1 — Figure S1 [file MBT2-16-1505-s004.docx]

**
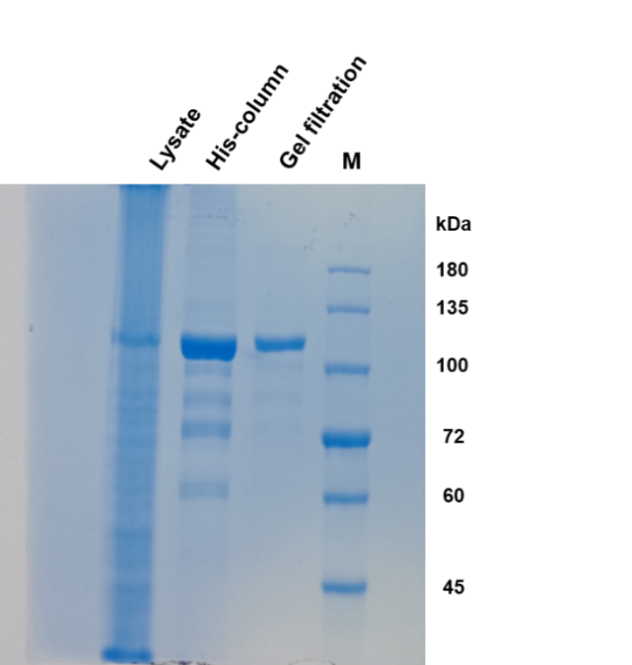
**

Supplementary Figure S1. SDS polyacrylamide gel electrophoresis showing the steps of His-tagged EHCas9 purification. A whole lysate of bacteria expressing EHCas9 (Lysate) and samples of protein extracts purified through His-binding column (His-column) as well as after subsequent gel filtration (Gel filtration) are included. The size of bands corresponding to the protein molecular weight marker (M) is indicated. The major band of the protein extracts corresponds to a protein around 120 kDa as expected for the His-tagged EHCas9.
