# Supplementary material for: Identification of the EH CRISPR‐Cas9 system on a metagenome and its application to genome engineering
Source: Microb Biotechnol. 2023 Apr 25;16(7):1505–23. doi: 10.1111/1751-7915.14266 (PMC10281378; doi:10.1111/1751-7915.14266)
Supplement: Supplementary file 2 — Figure S2 [file MBT2-16-1505-s008.docx]

**A**

EHCas9 MTHNAYTLGLDIGTNSIGWCILGDQRI------QDIGVRIFSDGRDPKSKMSLAVDRRAARAMRRRRDR-YVRR--RMKLLNLLVEYGLLPLDKQERKALQVENPYQIRALA-----LDE

CdCas9 ---MKYHVGIDVGTFSVGLAAIEVDDAGMPIKTLSLVSHIHDSGLDPDEIKS-AVTRLASSGIARRTRRLYRRKRRRLQQLDKFIQRQGWPVIELEDY-SDPLYPWKVRAELAASYIADE

* * * ** * * * * ** * ** * * ** * * * * * * * * ** **

RuvCI

BH

EHCas9 ALPAFQIGRALFHLNQRRGFKSNRKTDKGDPESGKISSAVVKLD-TLMREAG-----SRTFGEFLWKRLQAGLPVRIRMRDGEGPEKKDGSRSDGYAFYPDRASLEAEFEAIWTAQAAHH

CdCas9 KERGEKLSVALRHIARHRGWRNPYAKVSSLYLPDGPSDAFKAIREEIKRASGQPVPETATVGQMVTLCELG----TLKL-RGEGGVLSARLQQSDYA---------REIQEICRMQE---

** * ** * * * * * * *** ** * * *

EHCas9 TEIMTAARKAHLFDVIFYQRPLKQP---DVGLCAFLGGETGERRLAKSDPLFQKRRLLEELNALTIERGPGMTPERLTPDQRDRLLM------------LLRGKKSVSFSSMRKTLKLGD

CdCas9 ---IGQELYRKIIDVVFAAESPKGSASSRVGKDPL---QPGKNRALKASDAFQRYRIAALIGNLRVR-----------VDGEKRILSVEEKNLVFDHLVNLTPKKEPEWVTIAEILG---

** * * ** * * * ** * * * * * ** *

REC

EHCas9 AVFNKERAGRDKLLGDEVFAELSNKTRFGPAWGEVPIDDQRRIVARLRDEQDGAKLVDWLMTECSLGEGRARAVAGARLPEHFGRTGETATRAIIRELSTH--------TISGKVCVYSE

CdCas9 -------IDRGQLIGTATMTDDGERAGA-----RPPTHDTNRSI----VNSRIAPLVDWWKTASALEQHAMVKALSNAEVDDFDSPEGAKVQAFFADLDDDVHAKLDSLHLPVGRAAYSE

* * * * * * **** * * * * * ***

EHCas9 AVANAPELKHHSDFRTGEIMDTLPYYGEVLD-RHIMPGTGDPGDPIEVRIG-KLTNPTVHIGLNQLRRLINQILKVHGHPKQVIVEIARDLKSSEDQKKDIQRRQKQDQQEAERRGRALL

CdCas9 DTLV--------RLTRRMLSDGVDLYTARLQEFGIEPSWTPP----TPRIGEPVGNPAVDRVLKTVSRWLESATKTWGAPERVIIEHVREGFVTEKRAREMDGDMRR---RAARNAKLFQ

* * * * * * *** ** * * * * * * ** * * * * *

RuvCII

EHCas9 ELGQ-PNTGANRALLKLWEELNPGNPLDRRCIYTGQMISPRMLFSGAVDVDHILPWSRTLDDSSANKLVCMSDANRQKRNQTPFEAWGGTADWDGILARASALPA-----------SKAM

CdCas9 EMQEKLNVQGKPSRADLWRYQS-VQRQNCQCAYCGSPIT-----FSNSEMDHIVPRAGQGSTNTRENLVAVCHRCNQSKGNTPFAIWAKNTSIEGVSVKEAVERTRHWVTDTGMRSTDFK

* * ** * * * * *** * ** * *** * *

HNH

EHCas9 RFNPDAMNRFDNE---GGFLARHLV-----DTQYLSRLAKTYLEAIAPDRVYVSTGHLTAMLRRHWGLNSLLPDHNFSKTVHEKNRLDHRHHAIDAAVVGVLTLGLIQRVSKAAGQRELA

CdCas9 KFTKAVVERFQRATMDEEIDARSMESVAWMANELRSRVAQHFASHGT--TVRVYRGSLTAEARRASGISGKLK---FF-DGVGKSRLDRRHHAIDAAVIAFTSDYVAETLAVRS----NL

* ** ** ** * * * * *** ** * * * * *** *********

RuvCIII

EHCas9 GVQDVVDSIAPPWESFRDDLGR--------------VLAGLIVSHRPDHGTIGG-A-----RSKAFDQTAGRLHNDTAYGLTGETDTRANGLVVHRVSLAALKPSHLEPEGARIRDEEIR

CdCas9 KQSQAHRQEAPQWREFTGKDAEHRAAWRVWCQKMEKLSALLTEDLRDDRVVVMSNVRLRLGNGSAHKETIGKLSKVKL---S------------SQLSVSDID-----------------

** * * * * * * * * * * *

PLL

EHCas9 LALRKHTQGLDGKAFTDAVLDFARKDPKFKGMRA---------------VRVIEPLAVIPIKNGDGQAYKGYKGDAN--YRYDVWERPDGSWAA-DVISMFDAHSGDYESPIKRAYPTAR

CdCas9 ------------KASSEALWCALTREPGFDPKEGLPANPERHIRVNGTHVYAGDNIGLFPVSAGSIALRGGYAELGSSFHHARVYKITSGKKPAFAMLRVYTIDLLPYRNQD--LFS---

** * * * * * * ** * * * *

WED

EHCas9 KVLRLHQNDM-VRFDDEKAGDGIARVVKFGQSGSIYLAL----------------------HNESGALKA-------------------------RDSLP---QEDDPFKYLTASASRLK

CdCas9 --VELKPQTMSMRQAEKKLRDA----LATG--NAEYLGWLVVDDELVVDTSKIATDQVKAVEAELGTIRRWRVDGFFSPSKLRLRPLQMSKEGIKKESAPELSKIIDRPGWLPAVNKLFS

* * * * * * ** * * * * * * *

PI

EHCas9 NAKARQVYIDEIGRIRDPGPRTRP------

CdCas9 DGNVTVVRRDSLGRVRLESTAHLPVTWKVQ

* * ** * *

**B**

EHCas9 -----MTHNAYTLGLDIGTNSIGWCILGDQ-------------------RIQDIGVRIFSDGRDPKSKMSL---AVDRRAARAMRRRRDRYVRRRMKLLNLLVEYGLLPLDKQ-------

**1**

**|**

CdCas9 --------MKYHVGIDVGTFSVGLAAIEVDDAGM-------------PIKTLSLVSHIHDSGLDPDEIKSAVTRLASSGIARRTRRLYRRKRRRLQQLDKFIQRQGWPVIELE-------

CjCas9 --------MARILAFDIGISSIGWAFSENDE-------------------LKDCGVRIFTKVENPKTGESL---ALPRRLARSARKRLARRKARLNHLKHLIANEFKLNYEDYQ-SFDES

Nm1Cas9 MAAFKPNSINYILGLDIGIASVGWAMVEIDEEEN-------------PIRLIDLGVRVFERAEVPKTGDSL---AMARRLARSVRRLTRRRAHRLLRTRRLLKREGVLQAANF----DE-

St1Cas9 -------MSDLVLGLDIGIGSVGVGILNKVTG-----------------EIIHKNSRIFPAAQAE-----N---NLVRRTNRQGRRLARRKKHRRVRLNRLFEESGLITDFT--------

SaCas9 ---GSHMKRNYILGLDIGITSVGYGIIDYETR-----------------DVIDAGVRLFKEANVE-----N---NEGRRSKRGARRLKRRRRHRIQRVKKLLFDYNLLTDHS--------

SpCas9 ------MDKKYSIGLDIGTNSVGWAVITDEYKVPSKKFKVLGNTDRHSIKKNLIGALLFDSGET----------AEATRLKRTARRRYTRRKNRICYLQEIFSNEMAKVDDSFFHRLEES

* * * * * * * *

EHCas9 --------------------ER-KALQVENPYQIRALAL-----DEALPAFQIGRALFHLNQRRGFKSNRKTDK--G-------------------D---PESGKISSAV----VKLDTL

**100**

**|**

CdCas9 -----------------------DYSDPLYPWKVRAELAASYIADEKERGEKLSVALRHIARHRGWRNPYAKVSSLY-----------------LPDGPSDAFKAIREEI----KRASGQ

CjCas9 L------------------AKA-YKGSLISPYELRFRAL-----NELLSKQDFARVILHIAKRRGYDDIKNS----D-------------------D---KEKGAILKAI----KQNEEK

Nm1Cas9 -------------------NGL-IKSLPNTPWQLRAAAL-----DRKLTPLEWSAVLLHLIKHRGYLSQRKNEGETA-------------------D---KELGALLKGV----AGNAHA

St1Cas9 -----------------------KISINLNPYQLRVKGL-----TDELSNEELFIALKNMVKHRGISYLDDASDD-G-------------------N---SSVGDYAQIV----KENSKQ

SaCas9 -----------------------EL-SGINPYEARVKGL-----SQKLSEEEFSAALLHLAKRRGVHNVNEVEED-T-------------------G---NELSTKEQ-I----SRNSKA

SpCas9 FLVEEDKKHERHPIFGNIVDEVAYHEKYPTIYHLRKKLVDS---TDKADLRLIYLALAHMIKFRGHFLIEGDLNPDNSDVDKLFIQLVQTYNQLFEENPINASGVDAKAILSARLSKSRR

* **

EHCas9 MR--------EAGSRTFGEFLWKRLQ-AGLPVRI----------RMRDGEG---------------------------------------------------------------------

CdCas9 P---------VPETATVGQMVTLCEL-G--------------------------------------------------------------------------------------------

CjCas9 LA----------NYQSVGEYLYKEYF-QKFKE---------------NSKE---------------------------------------------------------------------

Nm1Cas9 LQ--------------TGDFRTPAE--LALNK---------------FEKE---------------------------------------------------------------------

St1Cas9 LETKTPGQIQLERYQTYGQLRGDFTV-E--------------------------------------------------------------------------------------------

SaCas9 LEEKYVAELQLERLKKDGEVRG--------------------------------------------------------------------------------------------------

SpCas9 LEN---LIAQLPGEKKNGLFGNLIALSLGLTPNFKSNFDLAEDAKLQLSKDTYDDDLDNLLAQIGDQYADLFLAAKNLSDAILLSDILRVNTEITKAPLSASMIKRYDEHHQDLTLLKAL

*

**200**

**|**

EHCas9 -----------------------------------------PEKKDGSRS----------------DGYAFYPDRASLEAEFEAIWTAQAAHHTEIMTAARKAHLFD----VIFYQRPL-

CdCas9 -----------------------------------------TLKLRGEGG----------------VLSARL-QQSDYAREIQEICRMQE-IGQELYRKII----DV-----VFAAES--

CjCas9 -----------------------------------------FTNVRNKKE----------------SYERCI-AQSFLKDELKLIFKKQREFGFSFSKKF-EEEVLSV----AFYK----

Nm1Cas9 -----------------------------------------SGHIRNQRS----------------DYSHTF-SRKDLQAELILLFEKQKEFGNPHVSGGLKEGIETL----LMTQRP--

St1Cas9 --------------------------------------------KDGKKH----------------RLINVF-PTSAYRSEALRILQTQQEFNPQITDEFINRYLEILTGKRKYYHGPGN

SaCas9 -------------------------------------------------------------------SINRF-KTSDYVKEAKQLLKVQKAY-HQLDQSFIDTYIDLLETRRTYYEGPGE

SpCas9 VRQQLPEKYKEIFFDQSKNGYAGYIDGGASQEEFYKFIKPILEKMDGTEELLVKLNREDLLRKQRTFDNGSI-PHQIHLGELHAILRRQEDFYPFL-KDNREKIEKILTFRIPYYVGPLA

* *

EHCas9 -----------------------------------------KQPDVGLCAFLGGETGERRLAKSDPLFQKRRLLEELNALTIERGPGMTPERLTPDQRDRLLMLLRGK----KSVSFSSM

**300**

**|**

CdCas9 -------------------------------------PKGSASSRVGKDPLQPGK---NRALKASDAFQRYRIAALIGNLRVRVDGEKR--ILSVEEKNLVFDHLVNL-TPKKEPEWVTI

CjCas9 ---------------R---------------------ALKDFSHLVGNCSFFTDE---KRAPKNSPLAFMFVALTRIINLLNNLKNTEGI-LYTKDDLNALLNEVLKN----GTLTYKQT

Nm1Cas9 --------------AL---------------------SGDAVQKMLGHCTFEPAE---PKAAKNTYTAERFIWLTKLNNLRI-LEQGSER-PLTDTERATLMDEPYRK----SKLTYAQA

St1Cas9 EKSRTDYGRYRTSGET---------------------LDNIFGILIGKCTFYPDE---FRAAKASYTAQEFNLLNDLNNLTVPTETK-KL---SKEQKNQIINYVKNEKAMGPAKLFKYI

SaCas9 GSPF---G-----WKD---------------------IKEWYEMLMGHCTYFPEE---LRSVKYAYNADLYNALNDLNNLVITRDENEKL---EYYEKFQIIENVFKQ---KKKPTLKQI

SpCas9 RG------NSRFAWMTRKSEETITPWNFEEVVDKGASAQSFIERMTNFDKNLPNE---KVLPKHSLLYEYFTVYNELTKVKYVTEGMRKPAFLSGEQKKAIVDLLFKT---NRKVTVKQL

*

EHCas9 RKT----LKLGDAVF-NKERAGRDKLLGDEVFAELSNKTRFGPAWGEVPIDDQRRIVARLRDEQDGAKLVDWLMTECSLGEGRARAVAGA-RLPEHFGRTGETATRAIIRELS-------

**400**

**|**

CdCas9 AEI----LGIDRGQLI-----------GTATMTDDGER-----AGARPPTHDTNRSIV----NSRIAPLVDWWKTASALEQHAMVKALSNAEVDDFDSPEGA-KVQAFFADLDD----DV

CjCas9 KKL----LGLSDDYEFKGEK--------GTYFIEFKKYKEFIKALGEHNLS---------QD------DLNEIAKDITLIKDEIK--LKK-ALAKYD------LNQNQIDSLS-------

Nm1Cas9 RKL----LGLEDTAFFKGLRYGKDN-AEASTLMEMKAYHAISRALEKEGLKDKKSPLNLSPE------LQDEIGTAFSLFKTDED--ITG-RLKDRI------QPEILEALLK-------

St1Cas9 AKL----LS-CDVADIKGYRIDKS---GKAEIHTFEAYRKMKTLET-------LDIEQMDRE------TLDKLAYVLTLNTEREG--IQE-ALEHEFA-DGS-FSQKQVDELVQFRKANS

SaCas9 AKE----IL-VNEEDIKGYRVTST---GKPEFTNLKVYHDIKDITA-------RKEIIENAE------LLDQIAKILTIYQSSED--IQE-ELTNLN---SE-LTQEEIEQISNL-----

SpCas9 KEDYFKKIECFDSVEISGV--------EDRFNASLGTYHDLLKIIKDKDFLD----------NEENEDILEDIVLTLTLFEDREM--IEE-RLKTYAH----LFDDKVMKQLK-------

**500**

**|**

EHCas9 ------THTISGKVCVYSE--A--VANAP-ELKHHSDFRTGEIMD-----TLPYYGEVLDRHIM-PGTGDPGDPIEVRIGKLTNPTVHIGLNQLRRLINQILKVHG--HPKQVIVEIARD

CdCas9 HAKLDSLH-LPVGRAAYSEDTL--VRLTRRMLSDGVDLYTA---------RLQEFG-------IE-PSWTPP--TPRIGEPVGNPAVDRVLKTVSRWLESATKTWG--APERVIIEHVRE

CjCas9 KLEFKDHLNISFKA-------L--KLVTP-LMLEGKKYDEA---------CNELNLKVAI--NEDKKDFLPAFNETYYKDEVTNPVVLRAIKEYRKVLNALLKKYG--KVHKINIELARE

Nm1Cas9 HISFDKFVQISLKA-------L--RRIVP-LMEQGKRYDEA---------CAEIYGDHYGKKNTEEKIYLPP----IPADEIRNPVVLRALSQARKVINGVVRRYG--SPARIHIETARE

St1Cas9 SIFGKGWHNFSVKL-------M--MELIP-ELYETSEEQMT---------ILTRLGKQKTTSSSNKTKY---IDEKLLTEEIYNPVVAKSVRQAIKIVNAAIKEYG--DFDNIVIEMARE

SaCas9 -KGYTGTHNLSLKA-------I--NLILD-ELWHTNDNQIA---------IFNRLKLVPKKVDLSQQKE---IPTTLVDDFILSPVVKRSFIQSIKVINAIIKKYG--LPNDIIIELARE

SpCas9 RRRYTGWGRLSRKLINGIRDKQSGKTILDFLKSDG--FANRNFMQLIHDDSLTFKEDIQKAQVSGQGD----SLHEHIANLAGSPAIKKGILQTVKVVDELVKVMGRHKPENIVIEMARE

* * * *

EHCas9 LKSSEDQKKDIQRRQKQDQQEAERRGRALL----------ELGQPNTGANRALLKLWEELNPGNPLDRRCIYTGQMISPRMLF--SGAVDVDHILPWSRTLDDSSA-NKLVCMSDANRQK

**600**

**|**

CdCas9 GFVTEKRAREMDGDMRRR---A-ARNAKLF---QEMQEKLNVQGKP-----SRADLWRY-QSVQRQNCQCAYCGSPITFS-------NSEMDHIVPRAGQGSTNTRENLVAVCHRCNQSK

CjCas9 VGKNHSQRAKIEKEQNENYKAKKDAELECE----------KLGLKINSKNILKLRLFKE------QKEFCAYSGEKIKISDLQ-DEKMLEIDHIYPYSRSFDDSYM-NKVLVFTKQNQEK

Nm1Cas9 VGKSFKDRKEIEKRQEENRKDREKAAAKFRE------YFPNFVGEPKSKDILKLRLYEQ------QHGKCLYSGKEINLGRLN-EKGYVEIDHALPFSRTWDDSFN-NKVLVLGSENQNK

St1Cas9 TNEDDE-KKAIQKIQKANKDEKDAAMLKAANQYNGKAELPHSVFHGHKQLATKIRLWHQ------QGERCLYTGKTISIHDLINNSNQFEVDHILPLSITFDDSLA-NKVLVYATANQEK

SaCas9 KNSKDA-QKMINEMQKRNRQTNERIE--EI--------IRTTGKENAKYLIEKIKLHDM------QEGKCLYSLEAIPLEDLLNNPFNYEVDHIIPRSVSFDNSFN-NKVLVKQEENSKK

SpCas9 NQTTQKGQKNSRERMKRIEEGIKELGSQI---------LKEHPVENTQLQNEKLYLYYL------QNGRDMYVDQELDINRLS----DYDVDHIVPQSFLKDDSID-NKVLTRSDKNRGK

* * ** * * * *

**700**

**|**

EHCas9 RNQTPFEAWGGT---ADWDGILARA------------SALPASKAM-RFNPDAMNRF---DNEGGFLARHLVDTQYLSRLAKTYLEAI--------------------APDRVYVSTGHL

CdCas9 G-NTPFAIWAKNTSI---EGVSVKEAVERTRHWVTDTGMRST--DFKKFTKAVVERFQRATMDEEIDARSMESVAWMANELRSRVAQHFASHG-----------------TTVRVYRGSL

CjCas9 LNQTPFEAFGND--SAKWQKIEV-------LA-----KNLPTKKQKRILDKN--YKD---KEQKNFKDRNLNDTRYIARLVLNYTKDYLDFLPLSDDENTKLNDTQKGSKVHVEAKSGML

Nm1Cas9 GNQTPYEYFNGKDNSREWQEFKARV-----ET-----SRFPRSKKQRILLQK--F------DEDGFKERNLNDTRYVNRFLCQFVADRMRLTG-------------K-GKKRVFASNGQI

St1Cas9 GQRTPYQALDSMDDAWSFRELKAF---------VRESKTLSNKKKEYLLTEEDISKF---DVRKKFIERNLVDTRYASRVVLNALQEHFRAHK---------------IDTKVSVVRGQF

SaCas9 GNRTPFQYLSSSDSKISYETFKKHI-----LNLAKGKGRISKTKKEYLLEERDINRF---SVQKDFINRNLVDTRYATRGLMNLLRSYFRVNN---------------LDVKVKSINGGF

SpCas9 SDNVPSEEVVKKMK-NYWRQLLNAK--------------LITQRKFDNLTKAERGGL-SELDKAGFIKRQLVETRQITKHVAQILDSRMNTKY---DENDKLI-----REVKVITLKSKL

* * *

**800**

**|**

EHCas9 TAMLRRHWGLNSLLPDHNFSKTVHEKNRLDHRHHAIDAAVVGVLTLGLIQRVSKAA---G--------Q------RELAGVQD--VVDSIAPPWESFRDDLGRVLA--------------

CdCas9 TAEARRASGISGKLKFF---DG-VGKSRLDRRHHAIDAAVIAFTSDYVAETLAVRSNLKQ---SQAHRQ-EAPQWREFTGKD-----AEHRAAWRVWCQKM-------------------

CjCas9 TSALRHTWGFSAK-------------DRNNHLHHAIDAVIIAYANNSIVK---AFSDFKKEQESNSAEL--Y---AKKISELDYKNKRKFFEPFSGFRQK--------------------

Nm1Cas9 TNLLRGFWGLRKV-------------RAENDRHHALDAVVVACSTVAMQQ---KITRFVRYKEMNAFDGKTI---DKETG-EVLHQKTHFPQPWEFFAQEVMIRVFGKPDGKPEFEEADT

St1Cas9 TSQLRRHWGIEKT-------------RD-TYHHHAVDALIIAASS-QLNLWKKQKNTLVSYSEDQLLDIETG----ELISDDEY-KESVFKAPYQHFVDTLKSKEFED------------

SaCas9 TSFLRRKWKFKKE-------------RNKGYKHHAEDALIIANADFIFKEWKKLDKAKKV-MENQMFEEKQAESMPEIETEQEY-KEIFI-TPHQI----KHIKDFK-------------

SpCas9 VSDFRKDFQFYKV-------------REINNYHHAHDAYLNAVVGTALIKKYPKLESEFVYGDYKVYDV------RKMIAKSEQ-EIGKATAKYFFYSNIMN--FFKT------------

* *** **

EHCas9 ------------------------GLIVSHRPDHGTIGGARSKAFDQTAG------------------------------------------RLHN----DTA-YGLTG--ETDTRANGL

CdCas9 -EKLSALLTEDLRDDRVVVMSNVR--------LRLGNGSAHKETIGKLSKVKL-SSQLSVSD-------------------IDKASSEALWCALTREPGFDPK-EGLPANPERHIRVNGT

CjCas9 ------------------VLDKIDEIFVSKPERKKPSGALHEETFRK-------------------------------------------------------------------------

Nm1Cas9 LEKLRTLLAEKLSSRPEAVHEYVTPLFVSRAPNRKMSGQGHMETVKS-------------------------------------------------------------------------

St1Cas9 --------------------SILFSYQVDSKFNRKISDATIYAT----------------------------------------------------------------------------

SaCas9 --------------------DYKYSHRVDKKPNRELINDTLYST----------------------------------------------------------------------------

SpCas9 ---EITLANGE----------------IRKRPLIETNGETGEIVWDKGRDFATVRKVLSMPQVNIVKKTEVQTGGFSKESILPKRNSDKL---IARKKDWDPKKYGGFDSPT--------

**900**

**|**

EHCas9 VVHRVSLAALKPSHLEPEGARIRD-EEIRLALRKHTQGLDG----KAFTDAVLDFA----------R--------KDPKFKGMRAVRVIEPLAVI-----PIKNGDGQAYKGYKGDANY-

CdCas9 HVYAGDNIGLFPVS---AGSIALRGGYAELGSSFHHARVYKITSGKKPAFAMLRVYT---IDLLPYRNQDLFSVELKPQTMSMRQA------------------------------EKKL

CjCas9 ------------E---------------------------------------------------------------------------------------------EEF--YQS-YGGKE

Nm1Cas9 ------------AKRLDEGVSVLR-------------------------VPLTQLKLKDLEKMVNRE--------REPKLYEALKARLEAHKDD------PAKAFAEPFYKYDK-AGNRT

St1Cas9 -------------RQAKVGKDKAD-ETYVLGK---IKDIYT----QDGYDAFMKIYKKDKSKFLMYR--------HDPQTFEKVIEPILENYPNKQINEKGKEVPCNPFLK-YKEEHGYI

SaCas9 -------------RKDDKG------NTLIVNN---LNGLYD----KD-NDKLKKLINKSPEKLLMYH--------HDPQTYQKLKL-IMEQYGD----------EKNPLYKYYEETGNYL

SpCas9 VAYSV----LV-VAKVEKGKSK------KLKS---VKELLGITIME------RSSFEKNPIDFLEAK------------GYKEVKKDLIIK---------------LPKYSLFELENGRK

EHCas9 -------------------RYDVWERP--DG-------------------------SWAADVISMFDAHSGD-YESPIK------------------RAYP----------TARKVLRLH

CdCas9 RDALA---TGN-------AEYLGWLVV--DDELVVDTSKIATDQVKAVEAELGTIRRWRVDGFF----SPSKLRLRPLQMSKEGIKKESAPEL----SKIIDRPGWLPA---VN---KLF

CjCas9 GVLKA---L----ELGK------IRKV--NGKI------------------VKNGDMFRVDIFK--HKKTNKFYAVPIY--TMDFALKVLPNKAVARSKKGEIKDWILMDENYEFCFSLY

Nm1Cas9 QQVKA---V----RVEQVQKTGVWVRN--HNGI------------------ADNATMVRVDVFE----KGDKYYLVPIY--SWQVAKGILPDRAVVQGK--DEEDWQLIDDSFNFKFSLH

St1Cas9 RKYSK---KGNGPEIKSLKYYDSKLGN--HIDITPKD--------SNNKVVLQSVSPWRADVYF--NKTTGKYEILGLKYADLQFEKGTG-TYKISQEKYNDIKKKEGVDSDSEFKFTLY

SaCas9 TKYSK---KDNGPVIKKIKYYGNKLNA--HLDITDDYPN------SRNKVVKLSLKPYRFDVYL--DNGVY--KFVTVK--NLDVIKKEN-YYEVNSKAYEEAKKLKKISNQAEFIASFY

SpCas9 RMLASAGELQKGNELALPSKYVNFLYLASHYEKLKGSPED----------------NEQKQLFV----EQHKHYLDEIIEQISEF-----------------SKRVILADANLDKVLSAY

**1000**

**|**

EHCas9 QNDMVRFDDEKAGDGIARVVKFGQSGSIYLALHNESGALKARDSLPQEDDPFKYLTA------------------------SASRLKNA-KARQ--VYIDEIGRIRDPGPRT-RP-----

CdCas9 SD-----------------------GNVTV------------------------------------------------------------------VRRDSLGRVRLESTAHLPVTWKVQ

CjCas9 KDSLILIQTKDMQE-----PEFVY----YNAFTSSTVSLIVSK----HDNKFETLSKNQK-----I---LFKNANEKEVIAKSIGIQNLKVFEK--YIVSALGEVTKA-EFRQREDFKK-

Nm1Cas9 PNDLVEVITKKAR-------MFGY----FASCHRGTGNINIRI----HDLD---------------------HKIGKNGILEGIGVKTALSFQK--YQIDELGKEIRPCRLKKRPPVR--

St1Cas9 KNDLLLVKDTETKE-----QQLFR----FLSR-----TMPKQK----HYVELKPYDKQKF-EGGEALIKVLGNVANSGQCKKGLGKSN-ISIYK--VRTDVLGNQHIIKNEGDKPKLDF-

SaCas9 NNDLIKINGELYRV-----IGVNN----DLLN-----RIEVNM----IDITYREYLENMNDKRPPRII-------------KTIASKT-QSIKK--YSTDILGNLYEVKSKKHPQIIKKG

SpCas9 NKHR----DKPIREQAENIIHLFT-----L-T-----NLG-------APAAFKYFDTTIDRKRYTS---------TKEVLDATLIHQSITGLYETRIDLSQLGGD---------------

*

Supplementary Figure S2. Amino acid sequence alignment of Cas9 proteins. (A) Pairwise sequence alignment of EHCas9 with the closest structurally characterized orthologue from Corynebacterium diphtheriae (CdCas9; protein data bank ID: 6JOO). The boundaries of RuvC (RuvCI-III motifs), Bridge Helix (BH), recognition (REC), HNH, Phosphate Lock Loop (PLL), WED, and PAM-Interacting (PI) domains of CdCas9 are denoted by colored bars bellow the sequence. (B) Multiple sequence alignment of EHCas9 with structurally characterized orthologues: CjCas9, Campylobacter jejuni; NmCas9, Neisseria meningitidis 8013; StCas9, Streptococcus thermophilus LMD9; SaCas9, Staphylococcus aureus; SpCas9, Streptococcus pyogenes. Some of the EHCas9 amino acid positions are listed. The RuvC and HNH catalytic sites are red and blue shaded, respectively. In both panels, conserved positions are marked with an asterisk.
