# Supplementary material for: Identification of the EH CRISPR‐Cas9 system on a metagenome and its application to genome engineering
Source: Microb Biotechnol. 2023 Apr 25;16(7):1505–23. doi: 10.1111/1751-7915.14266 (PMC10281378; doi:10.1111/1751-7915.14266)
Supplement: Supplementary file 3 — Figure S3 [file MBT2-16-1505-s001.docx]

**B**

**A**


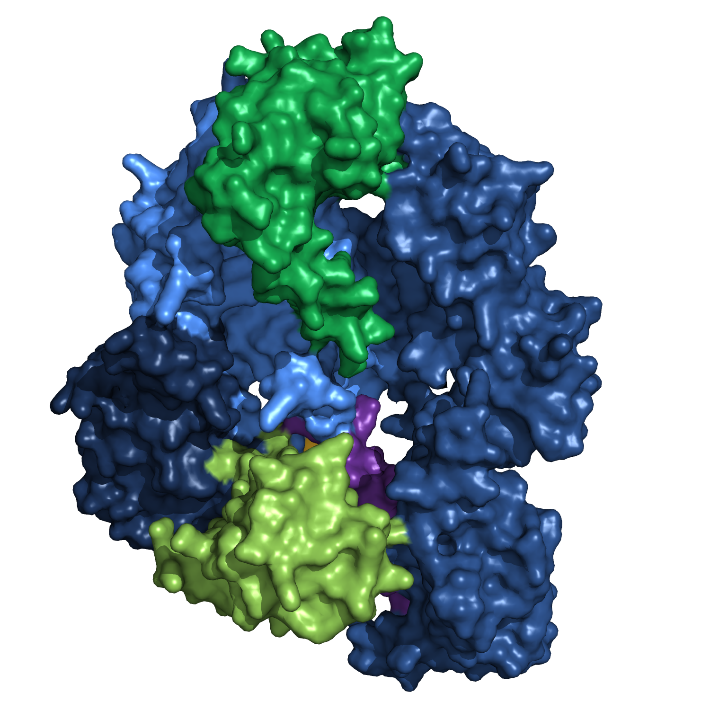

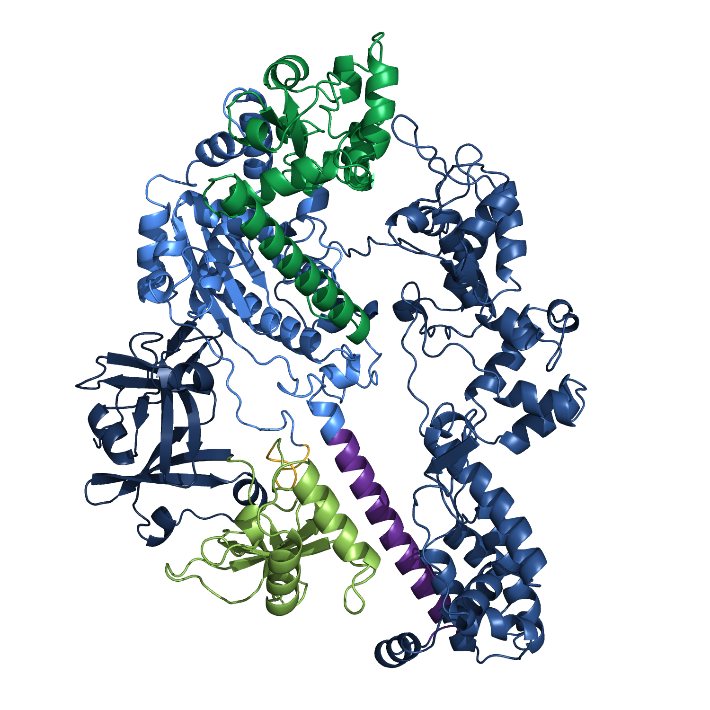

BH

HNH

REC

RuvC

PI

PLL

WEB

**C**

**D**


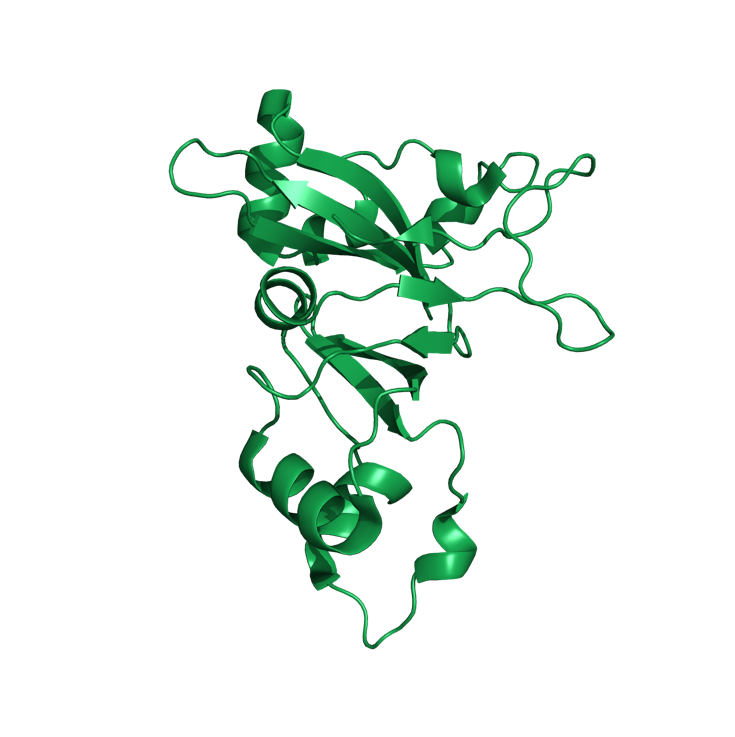

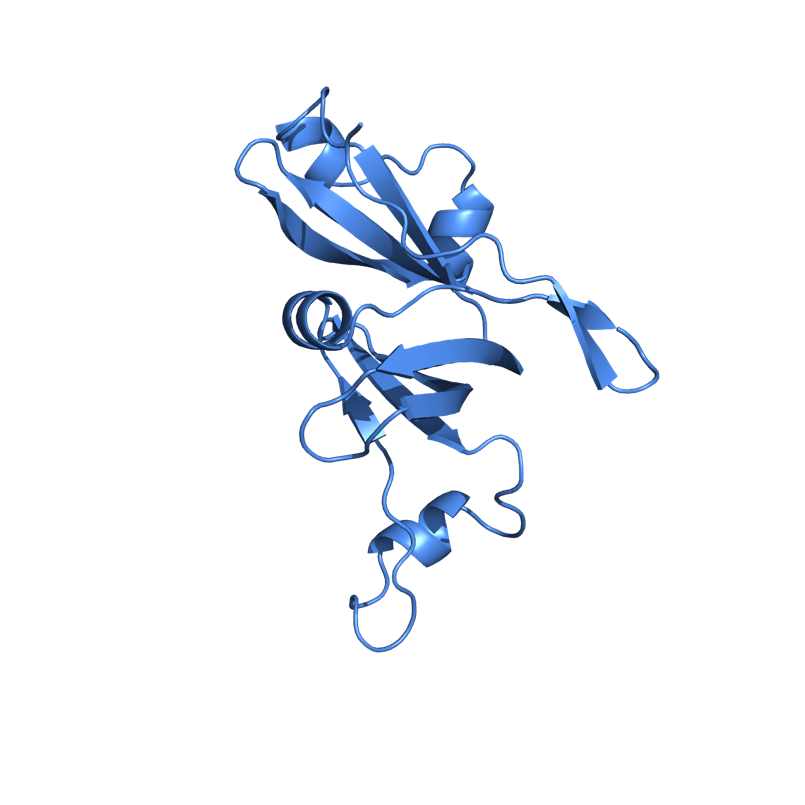


Supplementary Figure S3. EHCas9 predicted structures. (**A**) Cartoon representation of the EHCas9 3D structure predicted by AlphaFold2. α-helixes, β-sheets and loops are depicted. (**B**) Surface representation of the EHCas9 3D structure predicted by AlphaFold2. Protein domains are colored according to the legend. The typical bi-lobed structure of Cas9 can be observed. (**C**) Cristal structure of CdCas9 PI domain. (**D**) PI domain structure of EHCas9 predicted by AlphaFold2. The conserved core fold formed by antiparallel β-sheets that participate in PAM recognition can be observed.
