# Supplementary material for: Identification of the EH CRISPR‐Cas9 system on a metagenome and its application to genome engineering
Source: Microb Biotechnol. 2023 Apr 25;16(7):1505–23. doi: 10.1111/1751-7915.14266 (PMC10281378; doi:10.1111/1751-7915.14266)
Supplement: Supplementary file 4 — Figure S4 [file MBT2-16-1505-s002.docx]

******A B**

**C**

Supplementary Figure S4. Growth of N2a mouse cells expressing components of the EH and SpCas9 tools. (**A**) Total counts of nucleated cells in non-transfected cells (No plasmid) and after transfection with 200 ng, 150 ng and 100 ng of plasmids encoding SpCas9 or EHCas9. (**B**) Total counts of nuclei in non-transfected cells (No plasmid) and after transfection with 100 ng of plasmids encoding sgRNA of either SpCas9 (Sp sgRNA) or EHCas9 (EH sgRNA) (n=3, mean ± SD). (**C**) DAPI staining of non-transfected N2a cells (N2a) and cells after transfection with plasmids encoding SpCas9 or EHCas9.
