# Supplementary material for: Identification of the EH CRISPR‐Cas9 system on a metagenome and its application to genome engineering
Source: Microb Biotechnol. 2023 Apr 25;16(7):1505–23. doi: 10.1111/1751-7915.14266 (PMC10281378; doi:10.1111/1751-7915.14266)
Supplement: Supplementary file 5 — Table S1 [file MBT2-16-1505-s003.docx]

| Supplementary Table S1. Hidden Markov Models used in this work for the identification of Cas9 proteins in the metagenome dataset. | | |
| --- | --- | --- |
| **Accesion** | **Name** | **Description** |
| PF18470 | Cas9_a | Cas9 alpha-helical lobe domain |
| PF17893 | Cas9_b_hairpin | CRISPR-associated endonuclease Cas9 beta-hairpin domain |
| PF18525 | Cas9_C | Cas9 C-terminal domain |
| PF16595 | Cas9_PI | PAM-interacting domain of CRISPR-associated endonuclease Cas9 |
| PF18070 | Cas9_PI2 | CRISPR-Cas9 PI domain |
| PF16592 | Cas9_REC | REC lobe of CRISPR-associated endonuclease Cas9 |
| PF17894 | Cas9_Topo | Topo homology domain in CRISPR-associated endonuclease Cas9 |
| PF16593 | Cas9-BH | Bridge helix of CRISPR-associated endonuclease Cas9 |
| PF18061 | CRISPR_Cas9_WED | CRISPR-Cas9 WED domain |
| PF01844 | HNH | HNH endonuclease |
| PF13395 | HNH_4 | HNH endonuclease |
| PF14279 | HNH_5 | HNH endonuclease |
| PF02075 | RuvC | Crossover junction of endodeoxyribonuclease RuvC |
| PF18541 | RuvC_III | RuvC endonuclease subdomain 3 |
