# Supplementary material for: Identification of the EH CRISPR‐Cas9 system on a metagenome and its application to genome engineering
Source: Microb Biotechnol. 2023 Apr 25;16(7):1505–23. doi: 10.1111/1751-7915.14266 (PMC10281378; doi:10.1111/1751-7915.14266)
Supplement: Supplementary file 6 — Table S2 [file MBT2-16-1505-s006.doc]

| Supplementary Table S2. *E. coli* strains used in this work. | | | |
| --- | --- | --- | --- |
| **Strain** | **Relevant genotype** | **Use** | **Source/Reference^a^** |
| NZYstar | endA1 hsdR17(r_k_-, m_k_+) supE44 thi -1 recA1 gyrA96 relA1 lac[F´ proA^+^B^+^ lacI^q^ ZDM15 :Tn10(Tc^R^)] | Plasmid cloning and PAM library generation | NZYTech |
| BW 27783 | *lacIq rrnB3* Δ*lac*Z4787 Δ*(araBAD)*567 Δ(*rhaBAD*)568 *hsdR*514 Δ(K) Φ(*ΔaraEp* PCP8−*araE*) | *In vivo* PAM screening and validation. Positive selection of genome edited *E. coli* cells | 1 |
| TOP10 | *F- mcrA Δ(mrr-hsdRMS-mcrBC) φ80lacZΔM15 ΔlacX74 nupG recA1 araD139 Δ(ara-leu)7697 galE15 galK16 rpsL(StrR) endA1 λ-* | Cloning of plasmids used in eukaryotic genome editing | Invitrogen |
| BL21(DE3) | *F- ompT gal dcm lon* HsdSB (rB-mB-) λ(DE3 [*lacI lacUV5-T7* gene 1  *ind1 sam7 nin5])* | EHCas9 overexpression | 2 |

^a^References

1. Khlebnikov A, Datsenko KA, Skaug T, Wanner BL, Keasling JD. Microbiology. 2001;147: 3241–3247. doi:10.1099/00221287-147-12-3241

2. Rosenberg AH, Lade BN, Dao-shan C, Lin S-W, Dunn JJ, Studier FW. Gene. 1987;56: 125–135. doi:10.1016/0378-1119(87)90165-X
