# Supplementary material for: Identification of the EH CRISPR‐Cas9 system on a metagenome and its application to genome engineering
Source: Microb Biotechnol. 2023 Apr 25;16(7):1505–23. doi: 10.1111/1751-7915.14266 (PMC10281378; doi:10.1111/1751-7915.14266)
Supplement: Supplementary file 7 — Table S3 [file MBT2-16-1505-s005.doc]

| Supplementary Table S3. Plasmids used in this work. | | |
| --- | --- | --- |
| **Plasmid** | **Description** | **Reference^a^** |
| pBAD33 | Bacterial expression vector under arabinose promoter. Chloramphenicol resistance. | 1 |
| pUC57 | Bacterial cloning vector. Ampicillin resistance. | 2 |
| pSEVA431 | Bacterial cloning vector. Spectinomycin resistance. | 3 |
| pHTP1 | Bacterial expression vector under T7-lac promoter with a 6x histidine tag. Kanamycin resistance. | NZYTech |
| pKD46 | Temperature-sensitive replication bacterial plasmid encoding Lambda Red recombineering system. Ampicillin resistance. | 4 |
| hCas9 | Expression of humanized SpCas9 for genome engineering. Ampicillin and kanamycin resistance. | 5 |
| MLM3636 | SpCas9 sgRNA expression vector. Ampicillin resistance. | Keith Joung (unpublished) |
| pUC57-EHCas9 | pUC57 encoding EHCas9. | This work |
| pUC57-EHArray | pUC57 encoding EHCas9 CRISPR array. | This work |
| pMML01 | pBAD33-derivative containing EH CRISPR array. | This work |
| pMML02 | pMML01-derivative expressing EHCas9. | This work |
| pMML03 | pUC57-derivative containing EH CRISPR-Cas intergenic regions. | This work |
| pMML04 | pSEVA431-derivative with a target and 5’-TGGA-3’ PAM. | This work |
| pMML05 | pSEVA431-derivative with a target and 5’-TGGC-3’ PAM. | This work |
| pMML06 | pSEVA431-derivative with a target and 5’-TGGG-3’ PAM. | This work |
| pMML07 | pSEVA431-derivative with a target and 5’-TGGT-3’ PAM. | This work |
| pMML08 | pUC57-derivative containing EH sgRNA encoding sequence without spacer. | This work |
| pMML09 | pMML02-derivative by replacing CRISPR array with an EH sgRNA encoding sequence carrying a *pyrF* targeting spacer. | This work |
| pMML10 | pMML09-derivative by *ehcas9* deletion. | This work |
| pMML11 | pUC57-derivative carrying codon human optimized *ehcas9.* | This work |
| pMML12 | hCas9-derivative carrying codon human optimized *ehcas9* from pMML11 instead of *hcas9.* | This work |
| pMML13 | MLM3636-derivative encoding EH sgRNA (without spacer) from pMML08. | This work |
| pMML14 | MLM3636-derivative by insertion of a spacer targeting the *Oca2.*2 locus. | This work |
| pMML15 | MLM3636-derivative by insertion of a spacer targeting the *Oca2.*3 locus. | This work |
| pMML16 | MLM3636-derivative by insertion of a spacer targeting the *Oca2*.4 locus. | This work |
| pMML17 | MLM3636-derivative by insertion of a spacer targeting the *Lrmda.*1 locus. | This work |
| pMML18 | pMML13-derivative by insertion of a spacer targeting the *Oca2.*2 locus. | This work |
| pMML19 | pMML13-derivative by insertion of a spacer targeting the *Oca2.*3 locus. | This work |
| pMML20 | pMML13-derivative by insertion of a spacer targeting the *Oca2.*4 locus. | This work |
| pMML21 | pMML13-derivative by insertion of a spacer targeting the *Lrmda.*1 locus. | This work |
| pMML22 | pHTP1-derivative carrying *E. coli* codon optimized *ehcas9.* | This work |

^a^References

1. Guzman L-M, Belin D, Carson MJ, Beckwith J. J Bacteriol. 1995;177: 4121–4130. doi:10.1128/jb.177.14.4121-4130.1995

2. Yanisch-Perron C, Vieira J, Messing J. Gene. 1985;33: 103–119. doi:10.1016/0378-1119(85)90120-9

3. Silva-Rocha R, Martínez-García E, Calles B, Chavarría M, Arce-Rodríguez A, de Las Heras A, et al. Nucleic Acids Res. 2013;41: D666-D675. doi:10.1093/nar/gks1119

4. Datsenko KA, Wanner BL. Proc Natl. Acad Sci USA. 2000;97: 6640–6645. doi:10.1073/pnas.120163297

5. Mali P, Yang L, Esvelt KM, Aach J, Guell M, DiCarlo JE, et al. Science. 2013;339: 823–826. doi:10.1126/science.1232033
