# Supplementary material for: Identification of the EH CRISPR‐Cas9 system on a metagenome and its application to genome engineering
Source: Microb Biotechnol. 2023 Apr 25;16(7):1505–23. doi: 10.1111/1751-7915.14266 (PMC10281378; doi:10.1111/1751-7915.14266)
Supplement: Supplementary file 8 — Table S4 [file MBT2-16-1505-s007.docx]

Supplementary Table S4. Oligonucleotides used in this work.

| **Id.** | **Purpose** | **Target** | **Sequence (5’🡪 3’)** |
| --- | --- | --- | --- |
| 8 | pMML01 construction | pUC57-EHArray | GAAGCATTTATCAGGGTTATCGCACTCGAGTTTACAGCTAGC |
| 9 | pMML01 construction | pUC57-EHArray | ATGTATCCGCTCATGAGACATGCGCTCGAGAAAAAAAACCCCG |
| 10 | pMML01 construction | pBAD33 | TAGCTGTAAACTCGAGTGCGATAACCCTGATAAATGCTTCAATAATATTGAAAAAGGAAG |
| 11 | pMML01 construction | pBAD33 | GGTTTTTTTTCTCGAGCGCATGTCTCATGAGCGGATACATATTTGAATGT |
| 20 | pMML02 construction | pUC57-EHCas9 | TTTAAGAAGGAGATATACATATGACCCATAATGCATATACCTTGGG |
| 21 | pMML02 construction | pUC57-EHCas9 | GCCTGCAGGTCGACTCTAGATTACGGGCGCGTACGC |
| 22 | pMML02 construction | pMML01 | GTCCGCGTACGCGCCCGTAATCTAGAGTCGACCTGCAGGCATGCAAGCT |
| 23 | pMML02 construction | pMML01 | GTATATGCATTATGGGTCATATGTATATCTCCTTCTTAAAGGTACCGAGCT |
| 107 | Generation of pSEVA31-derived PAM library for *in vivo* PAM screening | pSEVA431 | TCATTATAGCGATTTTTTCNNNATATCCATCCTTTTTCGCACG |
| 108 | Generation of pSEVA31-derived PAM library for *in vivo* PAM screening | pSEVA431 | CGTGCGAAAAAGGATGGATATNNNGAAAAAATCGCTATAATGA |
| 159 | pMML04-07 construction | pSEVA431 | CCCCGAAGCAGGGTTATG |
| 134 | pMML04 construction | pSEVA431 | TCATTATAGCGATTTTTTCTCCATATCCATCCTTTTTCGCACG |
| 133 | pMML05 construction | pSEVA431 | TCATTATAGCGATTTTTTCGCCATATCCATCCTTTTTCGCACG |
| 187 | pMML06 construction | pSEVA431 | TCATTATAGCGATTTTTTCCCCATATCCATCCTTTTTCGCACG |
| 186 | pMML07 construction | pSEVA431 | TCATTATAGCGATTTTTTCACCATATCCATCCTTTTTCGCACG |
| 70 | Amplification of the PAM region for sequencing (PAM screening) | pSEVA431 | GCGAATAAGGGACAGTGAA |
| 60 | Amplification of the PAM region for sequencing (PAM screening) | pSEVA431 | GGCCCAGTATCAGCCCGTC |
| 169 | Generation of EH sgRNA dsDNA template for *in vitro* transcription (cleavage assays) | pMML08 | AAGCTAATACGACTCACTATAGGGTATCGTGCGAAAAAGGATGGATAGCTGCGGCTGGACCACGA |
| 170 | Generation of EH sgRNA dsDNA template for *in vitro* transcription (cleavage assays) | pMML08 | ACAAAAGAGGGCAAGGC |
| 115 | Generation of the dsDNA target for cleavage assays | pSEVA431 | ATTCTTGCAGGTATCTTCGAGCC |
| 15 | Generation of the dsDNA target for cleavage assays | pSEVA431 | CCAGCCTTGACCGAAACG |
| 218 | pMML09 construction | pMML08 | CTGCTGCAGCTGACTTAGGCGTGGCTGCGGCTGGACCACGA |
| 170 | pMML09 construction | pMML08 | ACAAAAGAGGGCAAGGC |
| 203 | Generation of dsDNA intermediate for pMML09 construction | pMML02 | TTTTTTTTTTATTATTATTAACAAAAGAGGGCAAGGCCG |
| 219 | Generation of dsDNA intermediate for pMML09 construction | pMML02 | ATTATGCTAGCATCCTAGATCTGCTGCAGCTGACTTAGG |
| 224 | pMML09 construction | dsDNA intermediate for pMML09 construction | CACGCCTAAGTCAGCTGCAGCAGATCTAGGATGCTAGCATAATACCTAGGACT |
| 202 | pMML09 construction | dsDNA intermediate for pMML09 construction | CCGGCCTTGCCCTCTTTTGTTAATAATAATAAAAAAAAAAACCCCGCCCCTGACAGGGC |
| 22 | pMML10 construction | pMML09 | GTCCGCGTACGCGCCCGTAATCTAGAGTCGACCTGCAGGCATGCAAGCT |
| 23 | pMML10 construction | pMML09 | GTATATGCATTATGGGTCATATGTATATCTCCTTCTTAAAGGTACCGAGCT |
| 188 | Generation of dsDNA template for recombination | *E. coli* BW 27783 | TCCATTGTGCTCCGGCAAC |
| 189 | Generation of dsDNA template for recombination | *E. coli* BW 27783 | CTGTTCTGGCGTCATAATGCGACCAGACCTTCTTGATGATGG |
| 190 | Generation of dsDNA template for recombination | *E. coli* BW 27783 | ATCATCAAGAAGGTCTGGTCGCATTATGACGCCAGAACA |
| 191 | Generation of dsDNA template for recombination | *E. coli* BW 27783 | TGAGTAGACCAGACGGCTG |
| 234 | Positive selection screening of *pyrF* gene | Colonies derived from genome edited *E. coli* cells | TACTGGCATTGTCCGTCTTGTC |
| 235 | Positive selection screening of *pyrF* gene | Colonies derived from genome edited *E. coli* cells | GCACCACACCGTCGCCT |
| P1 | pMML12 construction | pMML11 | TAGAGGATCGAACCCTTGCCACCATGGCCCACAATGCTTATACGC |
| P2 | pMML12 construction | pMML11 | TTCTTCTTGGGGTCAGCCCTGCTTGGGCGCGTTCTGGGAC |
| P3 | pMML12 construction | hCas9 | GATCCGGGTCCCAGAACGCGCCCAAGCAGGGCTGACCCCAAG |
| P4 | pMML12 construction | hCas9 | GCGTATAAGCATTGTGGGCCATGGTGGCAAGGGTTCGATCCTC |
| P5 | pMML13 construction | pMML08 | ACCGAGACGATTAATGCGTCTCGCTGCGGCTGGACCACGAG |
| P6 | pMML13 construction | pMML08 | CGAGCGGCCCAAGCTTAAAAAAAACAAAAGAGGGCAAGGCCGGAG |
| P7 | pMML13 construction | MLM3636 | GCTCCGGCCTTGCCCTCTTTTGTTTTTTTTAAGCTTGGGCCGCTCG |
| P8 | pMML13 construction | MLM3636 | GATTTCTCGTGGTCCAGCCGCAGCGAGACGCATTAATCGTCTCGG |
| P17 | Construction of pMML15 and pMML19 by Golden Gate | P18 (pMML15) and P19 (pMML19) | ACACCTACCAAAGGCTGCTGTGTGGAGAG |
| P18 | pMML15 construction by Golden Gate cloning | P17 | GCAGCTCTCCACACAGCAGCCTTTGGTAG |
| P19 | pMML19 construction by Golden Gate cloning | P17 | AAAACTCTCCACACAGCAGCCTTTGGTAG |
| P20 | Construction of pMML14 and pMML18 by Golden Gate cloning | P20 (pMML14) and P21 (pMML18) | ACACCGCTTGTCAAGTGCCTGACGGTGCG |
| P21 | pMML14 construction by Golden Gate cloning | P20 | GCAGCGCACCGTCAGGCACTTGACAAGCG |
| P22 | pMML18 construction by Golden Gate cloning | P20 | AAAACGCACCGTCAGGCACTTGACAAGCG |
| P23 | Construction of pMML17 and pMML21 by Golden Gate cloning | P24 (pMML17) and P25 (pMML21) | ACACCCAGAACTGGGAATAGTTTGGGCTG |
| P24 | pMML17 construction by Golden Gate cloning | P23 | GCAGCAGCCCAAACTATTCCCAGTTCTGG |
| P25 | pMML21 construction by Golden Gate cloning | P23 | AAAACAGCCCAAACTATTCCCAGTTCTGG |
| P26 | Construction of pMML16 and pMML20 by Golden Gate cloning | P27 (pMML16) and P28 (pMML20) | ACACCATTTGGTGGGTCCCCAATAGCAGG |
| P27 | pMML16 construction by Golden Gate cloning | P26 | GCAGCCTGCTATTGGGGACCCACCAAATG |
| P28 | pMML20 construction by Golden Gate cloning | P26 | AAAACCTGCTATTGGGGACCCACCAAATG |
| P45 | Amplification of *Oca2.*2 target site in genome editing assays | N2a cells from genome editing experiments | GGAAACACTTATGGTGATTCGAG |
| P46 | Amplification of *Oca2.*3 target site in genome editing assays | N2a cells from genome editing experiments | GCACAGGATTTCAGACAGG |
| P47 | Amplification of *Lrmda.*1 target site in genome editing assays | N2a cells from genome editing experiments | CGTGAAGGATGCGGTGAGTG |
| P48 | Amplification of *Oca2.*4 target site in genome editing assays | N2a cells from genome editing experiments | ATTTGGGTTGTGGACGGACC |
| P49 | Amplification of *Oca2.*2 target site in genome editing assays | N2a cells from genome editing experiments | GCAGCCTTTGGTACAGTTATC |
| P50 | Amplification of *Oca2.*3 target site in genome editing assays | N2a cells from genome editing experiments | CCCTTCTCACATTGAGCCCT |
| P51 | Amplification of *Lrmda.*1 target site in genome editing assays | N2a cells from genome editing experiments | GTCGTCTCCGAGCAGATTG |
| P52 | Amplification of *Oca2.*4 target site in genome editing assays | N2a cells from genome editing experiments | CCATCACGTTACTGTCAATACAGG |
