# Supplementary material for: Identification of the EH CRISPR‐Cas9 system on a metagenome and its application to genome engineering
Source: Microb Biotechnol. 2023 Apr 25;16(7):1505–23. doi: 10.1111/1751-7915.14266 (PMC10281378; doi:10.1111/1751-7915.14266)
Supplement: Supplementary file 9 — Table S5 [file MBT2-16-1505-s009.docx]

| Supplementary Table S5. Cas9 target sequences used in this work. | | |
| --- | --- | --- |
| **Experiment** | **Spacer-matching strand sequence**  **(5’ 🡪 3’)** | **PAM region**  **(5’ 🡪 3’)** |
| *In vivo* PAM screening | CCTGTATATCGTGCGAAAAAGGATGGATA | TNNNGAA |
| *In vivo* PAM validation | CCTGTATATCGTGCGAAAAAGGATGGATA | TACCGAA |
| *In vivo* PAM validation | CCTGTATATCGTGCGAAAAAGGATGGATA | TGGAGAA |
| *In vivo* PAM validation | CCTGTATATCGTGCGAAAAAGGATGGATA | TGGCGAA |
| *In vivo* PAM validation | CCTGTATATCGTGCGAAAAAGGATGGATA | TGGGGAA |
| *In vivo* PAM validation | CCTGTATATCGTGCGAAAAAGGATGGATA | TGGTGAA |
| *In vitro* PAM screening | GCTCGCTAAAGAGGAAGAGGACA | NNNNNNN |
| *In vitro* cleavage activity | TATCGTGCGAAAAAGGATGGATA | TACCGAA |
| *In vitro* cleavage activity | TATCGTGCGAAAAAGGATGGATA | TGGCGAA |
| Positive selection of edited *E. coli* cells | CTGCTGCAGCTGACTTAGGCGTG | TGGATGG |
| *Lrmda.*1 editing | CAGAACTGGGAATAGTTTGGGCT | TGGTGCC |
| *Oca 2.*2 editing | GCTTGTCAAGTGCCTGACGGTGC | TGGGATT |
| *Oca 2.*3 editing | TACCAAAGGCTGCTGTGTGGAGA | TGGATGT |
| *Oca 2.*4 editing | ATTTGGTGGGTCCCCAATAGCAG | TGGCAGC |
